# 原发纵隔大B细胞淋巴瘤诊断与治疗中国专家共识（2024年版）

**DOI:** 10.3760/cma.j.cn121090-20231107-00252

**Published:** 2024-03

**Authors:** 中华医学会血液学分会淋巴细胞疾病学组 中国临床肿瘤学会（CSCO）淋巴瘤专家委员会

**Keywords:** 淋巴瘤，大B细胞, 纵隔, 诊断, 治疗, Lymphoma, Large B-Cell, Mediastinum, Diagnosis, Therapy

## Abstract

原发纵隔大B细胞淋巴瘤（primary mediastinal large B-cell lymphoma, PMBL）是一种起源于纵隔的成熟侵袭性大B细胞淋巴瘤, 具有独特的临床、病理及分子学特征。近年来，对PMBL发病机制的认识及治疗均有不同程度的更新，特别是新药治疗领域取得了较大进展。为提高我国临床医师对PMBL的诊断及治疗水平，中华医学会血液学分会淋巴细胞疾病学组和中国临床肿瘤学会（CSCO）淋巴瘤专家委员会组织相关专家组，制订了本共识。

## Abstract (en)

Primary mediastinal large B-cell lymphoma (PMBL) is an aggressive B-cell lymphoma that is thought to arise from thymic (medullary) B cells and has unique clinicopathologic and molecular features. In recent years, the understanding of the pathogenesis and treatment of PMBL has been updated to varying degrees, particularly in the area of new drug therapy. In order to improve the diagnosis and treatment of PMBL in China, the Lymphocyte Disease Group of the Chinese Medical Association (CMA) and the Anti-Lymphoma Alliance of the Chinese Society of Clinical Oncology (CSCO) commissioned a group of experts to formulate this consensus.

原发纵隔大B细胞淋巴瘤（primary mediastinal large B cell lymphoma，PMBL）是一种起源于纵隔的成熟侵袭性大B细胞淋巴瘤，影像学上多为纵隔巨大肿物，而淋巴结和骨髓受累少见。因PMBL具有独特的临床、病理及分子学特征，2001年WHO造血与淋巴组织肿瘤分类将其明确归类为大B细胞淋巴瘤的一个独立亚型[1]并保留至今[2]。近年来，对PMBL发病机制的认识及治疗均有不同程度的更新，特别是新药治疗领域取得了较大进展。为提高我国临床医师对PMBL的诊断及治疗水平，中华医学会血液病学分会淋巴细胞疾病学组和中国临床肿瘤学会（CSCO）淋巴瘤专家委员会组织相关专家组根据国际上相关指南[3]–[4]及循证医学研究结果，讨论并制定本共识。

一、概述

根据中国淋巴瘤病理研究协作组发布的数据，我国PMBL的患者占B细胞非霍奇金淋巴瘤比例约为3.74％，占大B细胞淋巴瘤比例约为6.94％[5]，与西方国家类似[6]。基于美国国家癌症研究所SEER数据库分析表明，PMBL发病率有逐年增加的趋势[7]，女性居多，男女比例为1∶2，诊断时中位年龄约为35岁。初诊患者病灶多局限于胸腔内，远处累及相对少见。国内一项回顾性研究显示，我国PMBL患者5年无进展生存（PFS）率及总生存（OS）率分别为69％和75％[8]，略低于欧美国家。

二、临床表现

临床症状和体征多与快速增长的纵隔肿块相关，肿块可局部浸润至锁骨上淋巴结，也可通过胸腔延伸至肺、胸壁、心包和胸膜间隙等部位。患者发病初期常无明显症状，当肿块增大致气管、食管、肺等组织器官受压时，可表现出相应的临床症状，如刺激性干咳、吞咽不适及胸闷气促等不适；当压迫上腔静脉时，可出现上腔静脉综合征，表现为颈面部、胸背部及上肢水肿。B症状包括发热、盗汗和体重减轻较为常见，胸腔积液和心包积液发生率约为30％。胸腔外部位累及在初诊时非常罕见，但在复发时常见。

三、诊断及鉴别诊断

PMBL属于临床病理诊断，需结合临床和病理特点。根据肿瘤特定基因表达谱在分子水平上做出诊断是未来方向[9]–[10]。

（一）病理诊断

1. 病理取材：病理检查是确诊依据，优先推荐病变部位切取活检。对于取材困难等情况，空芯粗针穿刺或经内镜活检也是可行方式，但需确保有足量标本组织。

2. 形态学特点：PMBL表现为肿瘤细胞弥漫性浸润，通常有不同程度的纤维化，从纤细的胶原纤维到致密宽阔的纤维间隔将瘤细胞包绕呈簇状或片状分布。肿瘤细胞表现出广泛的细胞学多样性，但通常为中等至大细胞形态，具有丰富淡染或透亮胞质，核圆形或卵圆形，可见核仁，常见核分裂象。部分病例可见多形性细胞、Hodgkin-Reed-Sternberg（HRS）细胞。背景中可见数量不等的反应性淋巴细胞、组织细胞和粒细胞。可伴局灶坏死，偶尔可见残留的胸腺上皮成分。

3. 免疫表型：肿瘤细胞表达CD45和B细胞谱系抗原，如CD19、CD22、CD20和CD79a。尽管存在免疫球蛋白基因的功能重排和B系转录调节因子如BOB1、PU.1、OCT2和PAX5的表达，但通常缺乏表面和细胞质免疫球蛋白表达。大多数PMBL（>80％）CD30阳性，但与经典型霍奇金淋巴瘤（CHL）不同的是，染色通常呈异质性且表达较弱。肿瘤细胞常（75％～95％）表达MUM1，可变性表达BCL6和BCL2，CD10表达不常见（<30％的病例）。在>70％的PMBL表达且对诊断具有高特异性和敏感性的生物标志物包括：CD23、MAL（Myelin and lymphocyte）、CD200、PD-L1和PD-L2[11]。罕见的病例CD15阳性，常呈核旁小点状表达。EBV通常阴性。

4. 分子学特征：PMBL具有独特的转录组学特征，不同于其他大B细胞淋巴瘤，但与CHL有相似之处，表现为JAK-STAT与NF-κB信号通路的过度激活和免疫逃逸增强[12]–[13]，机制除涉及9p24、2p16.1及18p21等区域扩增外，还与相关负调控基因突变导致功能缺失以及CIITA易位造成MHC Ⅱ类蛋白表达下调等有关[14]。

（二）鉴别诊断

PMBL的主要鉴别诊断包括非特指性弥漫性大B细胞淋巴瘤（DLBCL，NOS）累及胸腺、CHL及纵隔灰区淋巴瘤（MGZL）等。根据组织学形态和免疫表型，累及纵隔的DLBCL，NOS与PMBL的区分最具有挑战性。DLBCL，NOS可出现纵隔淋巴结受累，但通常伴有广泛的胸部外疾病，因此了解临床信息至关重要。活检中若见残余胸腺上皮则更支持诊断PMBL。免疫组化CD30、CD23、MAL、CD200、PD-L1/L2的表达有助于与DLBCL，NOS鉴别。在困难的情况下，辅助基因表达检测、CIITA（C2TA）基因重排/突变检测、CD274（PD-L1）和PDCD1LG2（PD-L2）拷贝数或基因突变（如SOCS1、STAT6）检测可有利于鉴别诊断。

根据目前观点，纵隔外出现的具有PMBL特征的大B细胞淋巴瘤病例应归类为具有不同表型和遗传特征的DLBCL，是否存在极为罕见的令人信服的“纵隔外PMBL”病例仍有待进一步研究[9]–[10],[15]。当出现明显纤维化和HRS细胞时，需要与CHL鉴别诊断。CD45和B细胞抗原如CD79a、CD19和转录因子的缺乏，以及CD30在所有肿瘤细胞中的强表达，支持诊断CHL而不是PMBL。EBV阳性有利于CHL的诊断，尽管可以在非常罕见的PMBL病例中观察到。虽然CHL和PMBL在基因表达谱上有很多相似之处，但MAL在CHL中的表达非常罕见，有助于鉴别两者。MGZL与PMBL的鉴别在穿刺活检标本中很困难，常需要切取活检。与PMBL相反，具有PMBL样形态的MGZL CD30常一致性强阳性，B细胞转录因子OCT2、BOB1部分或完全丧失和（或）CD15强表达。

四、临床分期及预后评估

建议^18^F-FDG PET-CT作为初诊时PMBL患者分期的检查手段，推荐使用2014年Lugano修订版Ann Arbor分期系统[16]。需要特别指出的是对于纵隔肿块直接蔓延累及周围器官者归入Ⅱ期。约80％患者初诊时临床分期为Ⅰ～Ⅱ期。

目前尚无成熟的预后模型，国际预后指数（IPI）系统对PMBCL患者的预后判断有限。回顾性研究显示不良预后因素包括：LDH水平>正常上限的2倍、年龄>40岁、男性、临床分期为进展期、与纵隔非连续性的结外侵犯（如肾脏、肾上腺、肝脏、卵巢）、诱导治疗效果差（<部分缓解）等[8],[17]。

五、治疗

治疗流程见图1。

**图1:**
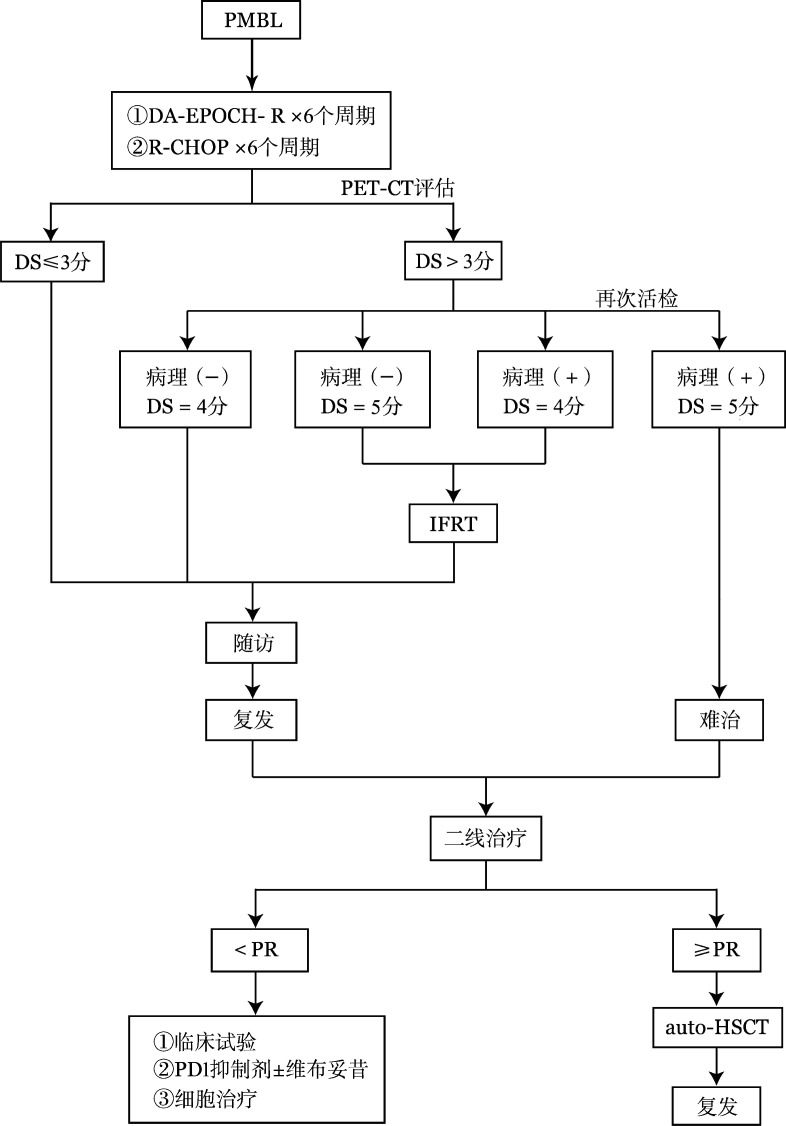
原发纵隔大B细胞淋巴瘤（PMBL）患者治疗流程图 注 DA-EPOCH-R：环磷酰胺+多柔比星+依托泊苷+长春新碱+泼尼松+利妥昔单抗；R-CHOP：利妥昔单抗+环磷酰胺+多柔比星+长春新碱+泼尼松；PET-CT：正电子发射断层扫描/X射线计算机断层成像；DS：多维尔评分；IFRT：受累野放射治疗；PR：部分缓解；auto-HSCT：自体造血干细胞移植；PD1：程序性死亡受体1

（一）治疗前评估

治疗前（包括复发患者）应对患者进行全面评估，参考其他类型淋巴瘤，包括病史和体格检查、体能状态评分、化验检测、影像学检查（推荐全身^18^F-FDG PET-CT检查或颈、胸、全腹部增强CT检查）、心脏彩超（左室射血分数）或多门控探测（MUGA）扫描等。

（二）初治患者治疗原则

随着利妥昔单抗的普及、^18^F-FDG PET-CT驱动的分层治疗及支持治疗的进步使得PMBL患者的生存获得极大改善，5年OS率超过80％[18]，晚期复发并不常见[7]。因此，治疗方案的选择必须同时兼顾疗效和治疗相关的近远期不良反应。PMBL发病率较低，大样本的前瞻随机临床研究较少，目前尚无标准的治疗方案，治疗推荐依据以回顾性研究为主。

国内外研究均证实，PMBL患者一线接受抗CD20单抗联合CHOP方案（R-CHOP）治疗，2年PFS率和OS率分别为80％和90％以上[19]–[20]。值得指出的是，约70％的患者后期序贯局部放疗。为减少放疗相关的近远期不良反应，2013年报道一项单臂Ⅱ期临床研究，初治 PMBL患者接受DA-EPOCH-R治疗 6～8个周期，中位随访 63个月，5年无事件生存（EFS）率和OS率分别为 93％和97％[21]。真实世界的数据也证实PMBL 患者采用R-CHOP 序贯放疗或DA-EPOCH-R获得相似的疗效，5年PFS率分别为91.3％和92.6％[22]。因此本共识推荐R-CHOP或DA-EPOCH作为PMBL 患者一线治疗选择。

因CT无法准确判断化疗后残存肿物的性质，建议采用^18^F-FDG PET-CT进行疗效评估，参考Deauville五分法标准（5-PS）评分[23]，指导后期个体化分层巩固治疗，达到疗效和不良反应的平衡。获得完全代谢缓解（CMR，DS≤3分）的患者，不推荐常规行受累野放疗[18],[24]–[25]。未获得CMR（DS>3分）患者（大约30％）决定治疗策略前，优先推荐^18^F-FDG PET-CT引导下高代谢部位穿刺活检病理诊断，明确残留病灶性质。对于DS评分4分的患者，如果病理检查可见肿瘤细胞，则建议行受累野放疗（30 Gy），达到根治性放疗的目的；相反，如果病理检查未见肿瘤细胞，可考虑密切随访。DS评分5分且病理检查可见肿瘤细胞的患者预后差，放疗的地位尚不明确，优先推荐参加临床试验，或采取二线治疗方案；对于DS评分5分，而病理检查未见肿瘤细胞的患者，因目前资料有限，可酌情采取受累野放疗（30 Gy）或密切随访。具体治疗路径详见图1。

（三）难治/复发（R/R）PMBL的治疗

复发大多发生于治疗结束后2年内。R/R PMBL患者建议采用类似DLBCL的二线挽救性化疗方案，获得部分缓解（PR）以上疗效者序贯auto-HSCT巩固治疗，3年OS率和EFS率分别约为68％和65％[26]。对于二线治疗无效或进展的患者，可选择以下治疗。

1. 临床试验。

2. PD1抑制剂单药或联合治疗：KEYNOTE-170研究显示帕博利珠单抗治疗二线治疗失败R/R PMBL患者，ORR为41.5％，其中CR率为20.8％；中位随访48.7个月，4年OS率和PFS率分别为45.3％和33.0％。主要不良反应是中性粒细胞减少症、虚弱和甲状腺功能减退[27]。CheckMate 436研究显示纳武利尤单抗联合维布妥昔单抗治疗经二线以上治疗后R/R PMBL患者，中位随访时间为39.6个月，研究者评估ORR为73.3％（CR率为40.0％），2年PFS率和OS 率分别为55.5％ 和75.5％，中位PFS期为26个月，中位OS期未达到[28]。

3. 细胞治疗：CAR-T细胞治疗二线以上R/R PMBL患者也取得较好的疗效，ORR为73％～84％，CR率为53％～54％，中位随访15.4个月，42％的患者疗效仍持续保持[29]–[30]。

多项回顾性研究显示，三线治疗获得PR以上疗效且体能状态良好的患者，序贯异基因造血干细胞移植（allo-HSCT）巩固治疗，2年PFS率和OS率可达50.0％和58.0％[31]–[32]。因此，本共识建议allo-HSCT作为三线治疗有效患者的巩固治疗。

六、疗效评估和随访

疗效评价标准参照Lugano 2014标准[16]，建议采用^18^F-FDG PET-CT进行疗效评价。完成治疗后的前2年应每3个月进行1次随访，包括颈部、胸部及全腹部CT等。完成治疗后第3～5年每半年进行1次随访，每12个月进行1次CT检查。
